# Safety of infusing rituximab at a more rapid rate in patients with rheumatoid arthritis: results from the RATE-RA study

**DOI:** 10.1186/1471-2474-15-177

**Published:** 2014-05-24

**Authors:** Charles H Pritchard, Maria W Greenwald, Joel M Kremer, Norman B Gaylis, William Rigby, Steve Zlotnick, Carol Chung, Birgit Jaber, William Reiss

**Affiliations:** 1 Rheumatology Specialty Center, 2360 Maryland Road, Willow Grove, PA 19090, USA; 2 Desert Medical Advances, Palm Desert, CA, USA; 3 Albany Medical College and the Center of Rheumatology, Albany, NY, USA; 4 Arthritis & Rheumatic Disease Specialties, Aventura, FL, USA; 5 Geisel School of Medicine at Dartmouth, Hanover, NH, USA; 6 Genentech, Inc., South San Francisco, CA, USA; 7 F. Hoffmann-La Roche Ltd, Basel, Switzerland

**Keywords:** Rituximab, Rheumatoid arthritis, Infusion-related reactions, Adverse events

## Abstract

**Background:**

As recommended in the current prescribing information, rituximab infusions in patients with rheumatoid arthritis (RA) take 4.25 hours for the first infusion and 3.25 hours for subsequent infusions, which is a burden on patients and the health care system. We therefore evaluated the safety of infusing rituximab at a faster rate for an infusion period of 2 hours in patients with RA.

**Methods:**

Patients with an inadequate response to anti-TNF who were rituximab-naive or -experienced received 2 courses of rituximab: Infusion 1 (Day 1) was administered over the standard 4.25 hours, and Infusions 2 (Day 15), 3 (Day 168) and 4 (Day 182) were administered over a faster 2-hour period. The primary endpoint was incidence of infusion-related reactions (IRRs) associated with Infusion 2.

**Results:**

Of the 351 patients enrolled, 87% and 13% were rituximab-naive and -experienced, respectively. The incidence (95% CI) of IRRs associated with Infusion 1 was 16.2% (12.5%, 20.5%) and consistent with weighted historical incidence of 20.7% (19.4%, 22.1%). The incidence (95% CI) of IRRs associated with Infusions 2, 3, and 4 compared with respective weighted historical incidences at the standard infusion rate was 6.5% (4.1%, 9.7%) vs 8.1% (7.2%, 9.1%); 5.9% (3.5%, 9.3%) vs 11.5% (10.3%, 12.8%); and 0.7 (0.1%, 2.6%) vs 5.0% (4.2%, 6.0%), respectively. All IRRs were grade 1 or 2, except for 3 grade 3 IRRs associated with Infusion 1 and 2 grade 3 IRRs associated with Infusion 2. Four patients experienced a total of 5 grade 3 IRRs; 3 of these patients continued on to received subsequent infusions at the faster rate. There were no serious IRRs.

**Conclusion:**

This study demonstrated that rituximab can be administered at the faster infusion rate at the second and subsequent infusions without increasing the rate or severity of IRRs.

## Background

Rituximab, a chimeric monoclonal antibody that binds to the antigen CD20, is approved worldwide in combination with methotrexate (MTX) for the treatment of moderately to severely active rheumatoid arthritis (RA) in patients with an inadequate response to at least one tumor necrosis factor (TNF)-α inhibitors. In clinical studies, rituximab has been shown to improve the signs and symptoms of disease [1] and reduce the rate of joint damage progression [2], with sustained efficacy at 1 year upon retreatment [1,3,4].

The indicated dose of rituximab for the treatment of RA is two 1000-mg intravenous (IV) infusions separated by 2 weeks every 24 weeks or based on clinical evaluation, but not sooner than every 16 weeks. As recommended in the current prescribing information, rituximab infusions take 4.25 hours for the first infusion and 3.25 hours for the second and subsequent infusions [5].

The infusion regimen as currently recommended was based on the rituximab dosing regimen used to treat patients with non-Hodgkin’s lymphoma (NHL). The mechanism by which rituximab elicits infusion-related reactions (IRRs) is not well understood; however, there is evidence that symptoms may be associated with the release of inflammatory cytokines as a result of rituximab binding to CD20 on B cells and cell lysis [6,7]. Because patients with RA have a lower peripheral B cell burden at baseline compared with patients with lymphoma, this may partially explain why the incidence of IRRs reported in RA is lower than that in NHL [5].

The safety profile of rituximab has been documented in numerous clinical trials. IRRs are the most commonly reported adverse reaction with the majority occurring at the first infusion of the first course. In previously published randomized controlled trials of rituximab in RA, IRRs have been defined as an adverse event (AE) occurring during or within 24 hours of an infusion [5,8]. As reported by van Vollenhoven and colleagues in a pooled analysis of all-exposure patient (N = 3194) data from the RA global clinical trial program, the rate of IRRs was 23.0% during the first infusion of the first course and decreased with each subsequent infusion. Most IRRs were mild to moderate (grade 1 and 2) and rarely serious (0.5%) [8].

The burden of the rituximab standard infusion protocol on the health care system is not insignificant. Longer infusion times and frequent infusion rate changes result in longer observation times, increased nursing and administration staff workloads, and pose a temporal inconvenience to patients. Based on the success of rapid infusion protocols in the oncology setting [9,10], several studies have evaluated the practicality of accelerated rituximab infusion rates in patients with RA and found them to be safe and tolerable; however, these results are restricted by small study sizes and limited clinical details [11-16].

The objective of this prospective, multicenter study was to evaluate the safety of infusing rituximab at a faster rate for an infusion period of 2 hours.

## Methods

RATE-RA was a 30-week, prospective, multicenter, open-label, single-arm study conducted in 74 sites across the United States. Between July 26, 2011, and June 8, 2012, patients with moderately to severely active RA who had an inadequate response to at least 1 anti-TNF agent and were receiving concomitant MTX were enrolled.

Patients were eligible if they were ≥ 18 years of age, had RA for ≥ 6 months, and were either rituximab-naive or -experienced (defined as having received no more than 2 courses of rituximab 6 to 9 months prior to baseline, with each course consisting of 2 infusions of 1000 mg each). Use of MTX 10 to 25 mg/week (oral or parenteral) for at least 8 weeks prior to baseline was required, and concomitant corticosteroids (≤10 mg prednisone or equivalent) were allowed. Key exclusion criteria included a history of rheumatic autoimmune disease other than RA or secondary Sjögren syndrome; history of severe allergic or anaphylactic reactions to monoclonal antibodies; previous serious IRRs to any biologic therapy; grade 3/4 New York Heart Association congestive heart failure; history of significant arrhythmia; and evidence of serious uncontrolled hypertension.

This study was performed in accordance with the Declaration of Helsinki. The study was approved by the Copernicus Group Independent Review Board at all but four investigator sites, which received approval from their local governing institutional review board (Dartmouth-Hitchcock Medical Center – Committee for the Protection of Human Subjects; North Mississippi Health Services; Sutter Health and Western Institutional Review Board). All patients provided written informed consent (RATE-RA ClinicalTrials.gov identifier NCT01382940).

### Study design

**Figure 1:**
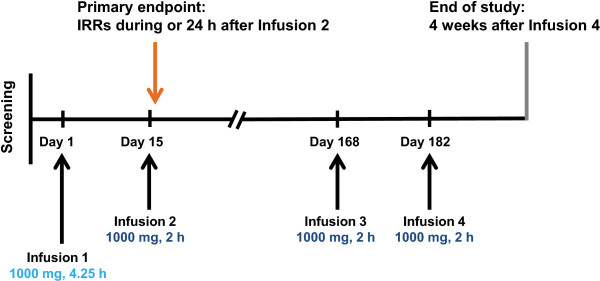
Study design.

The study design is summarized in Figure 1. Patients received 2 courses of rituximab 24 weeks apart, with each course consisting of two 1000-mg IV infusions of rituximab separated by 2 weeks. The first infusion was administered over the standard 4.25 hours while subsequent infusions were administered at faster rates for a 2-hour period. Infusion 1 (Day 1) was initiated at a rate of 50 mg/hour (Table 1). In the absence of infusion toxicity, the infusion rate was increased by 50 mg/hour increments every 30 minutes, to a maximum of 400 mg/hour. The total estimated time to complete a 1000-mg dose was 4 hours, 15 minutes (4.25 hours). For Infusions 2 (Day 15), 3 (Day 168) and 4 (Day 182), rituximab (1000 mg in 250 mL normal saline) was initiated at 250 mg/hour (Table 1). After 30 minutes, the rate was escalated to a rate of 600 mg/hour for the next 90 minutes. The total estimated time to complete a 1000-mg dose based on this rapid infusion schedule was just under 2 hours.

**Table 1:** Infusion schedule

**Time (min)**	**Infusion rate**		**Dose in 30 minutes**	**Cumulative dose**
	**(mg/h)**	**(mL/h)**	**(mg)**	**(mg)**
**Infusion 1**				
0-30	50	12.5	25	25
31-60	100	25	50	75
61-90	150	37.5	75	150
91-120	200	50	100	250
121-150	250	62.5	125	375
151-180	300	75	150	525
181-210	350	87.5	175	700
211-240	400	100	200	900
241-255	400	100	200	1000
**Infusion 2, 3 and 4**				
0-30	250	62.5	125	125
31-60	600	150	300	425
61-90	600	150	300	725
91 ≈ 120	600	150	275	1000

The total estimated time to complete a 1000-mg dose at Infusion 1 is 4 hours, 15 minutes (4.25 hours) with 8 infusion rate changes. The total estimated time to complete a 1000-mg dose (1000 mg diluted in 250 mL of normal saline or D5W) at Infusion 2, 3 and 4 is approximately 2 hours with 1 infusion rate change.

Premedication with 100 mg intravenous methylprednisolone, as well as 1 g acetaminophen and an antihistamine (diphenhydramine HCL 50 mg or equivalent dose of alternate) were administered orally 30 to 60 minutes prior to each rituximab infusion. Patients continued to receive MTX as prescribed by their treating physicians. Changes to MTX dose were allowed as long as the dose remained within 10 to 25 mg/week.

The following information was collected at each infusion: the initial infusion rate, the number and proportion of patients with an infusion modification and the reasons for modification; the number and proportion of patients who completed the 1000-mg dose; and the total infusion times among those who completed the 1000-mg dose.

### Safety assessments

All event incidences were calculated as per-patient incidences, defined as the proportion of patients experiencing the event. Multiple occurrences of a specific event for a patient were counted once. The primary endpoint was incidence of IRRs associated with Infusion 2. IRRs were defined as AEs that occurred during or within 24 hours of infusion. IRRs were identified based on a prespecified list of 180 Medical Dictionary for Regulatory Activities preferred terms, which has been used to analyze IRRs in patients with RA across rituximab studies conducted by Roche/Genentech. Secondary endpoints included incidence of serious IRRs associated with Infusion 2, incidence of IRRs and serious IRRs associated with Infusion 3, incidence of National Cancer Institute Common Toxicity Criteria (NCI-CTC) grade 3 or 4 AEs associated with Infusion 2 and 3, and incidence of stopping, slowing, or interrupting Infusion 2 and 3.

Exploratory analyses evaluated the aforementioned endpoints that were associated with Infusion 1 and 4. Subgroup analyses by patients’ previous exposure to rituximab were performed for all endpoints. Ad hoc analyses summarized the recurrence and severity of IRRs at subsequent infusions by the maximum NCI-CTCAE grade of IRRs at Infusions 1, 2, and 3.

### Statistical methods

This study aimed to have at least 300 patients receive the second rituximab infusion at a faster rate so that an increase of at least 3% to 4% of IRRs from the weighted reference incidence (8% to 9%, based on historical data for rituximab) would be statistically significant. A study size of 300 patients also provided 95% confidence to conclude that if there were no serious events, the incidence of serious IRRs was < 1%.

Integrated data from rituximab clinical development studies as of September 2012 were the basis for the historical incidences of IRRs associated with rituximab administration at the standard infusion rate. Incidences of IRRs were compared against the weighted historical incidences of IRRs, which were calculated using the historical incidences and adjusted for the proportion of patients with 0, 1, or 2 courses of prior rituximab to account for patients with prior rituximab who were more likely to have a lower IRR incidence.

Study data were summarized using descriptive statistics. The number and proportion of patients who experienced the respective endpoints were provided with a 2-sided 95% exact CI of the proportion. For serious IRRs, a 1-sided 95% exact confidence upper limit of the proportion was provided.

### Analysis populations

The safety evaluable population included all patients who received rituximab during the study and had an assessment during or after the infusion. AEs that occurred during the study were summarized based on the safety evaluable population. The faster infusion evaluable population included all patients who received Infusions 2, 3, or 4 at the faster rate at the respective visits (Day 15, 168, or 182, respectively). Patients were classified as having received a faster rate infusion for that visit if the infusion was completed within 2.5 hours or the infusion was not completed within 2.5 hours but started within 33% of the protocol-specified rate of 250 mg/hour (>167.5 mg/hour).

## Results

### Patient disposition

**Figure 2:**
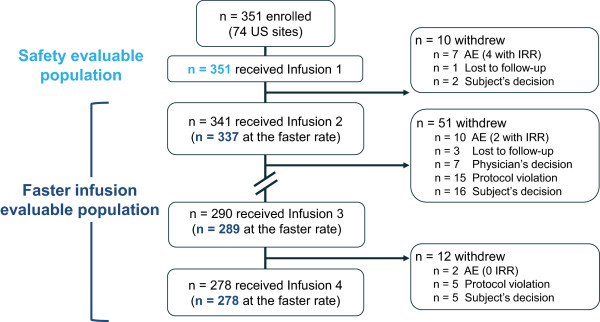
**Patient disposition.** IRR, infusion-related reaction.

The patient disposition is outlined in Figure 2. The number of withdrawals was consistent with that of previous trials of similar duration [1]. A total of 351 patients received ≥ 1 infusion of rituximab (safety evaluable population). Of the 341 patients who received Infusion 2, 337 patients were included in the faster infusion evaluable population. Four patients were excluded because infusion volume was not recorded. Of the 290 patients who received Infusion 3, 289 were included in the faster infusion evaluable population. One patient was excluded because the standard rate was used. All 278 patients who received Infusion 4 were included in the faster infusion evaluable population.

### Baseline demographics and clinical characteristics

The baseline demographics and clinical characteristics of the 337 patients in the faster infusion evaluable population were similar to the overall 351 patients in the safety evaluable population (Table 2). The mean duration of disease was approximately 12.5 years, and 50% had RA for more than 10 years. Approximately 87% (306/351) were rituximab-naive and 13% (45/351) were rituximab-experienced. Patients who were rituximab-naive and -experienced demonstrated similar baseline demographics (data not shown); however, rituximab- naive patients had a shorter mean disease duration by 2.4 years (12.2 ± 9.5 years vs 14.6 ± 11.1 years). Rituximab-naive patients demonstrated significantly higher mean CD19 counts compared with rituximab-experienced patients (252.6 ± 206.4 vs 47.9 ± 80.3 cells/μL); approximately 10% (31/306) and 82% (37/45) of rituximab-naive and -experienced patients, respectively, had CD19 counts below the lower limit of normal (<80 cells/μL).

**Table 2:** Baseline demographics and clinical characteristics

	**Safety population (n = 351)**	**Faster infusion evaluable population**^**a**^**(n = 337)**
**Demographics**		
Female, n (%)	279 (79.5)	268 (79.5)
Age, mean (SD), y	55.5 (11.5)	55.6 (11.4)
Range	23-88	23-88
Age group, n (%)		
18-40 y	38 (10.8)	34 (10.1)
41-64 y	244 (69.5)	236 (70.0)
≥ 65 y	69 (19.7)	67 (19.9)
Race, n (%)		
White	295 (84.0)	283 (84.0)
African American	35 (10.0)	33 (9.8)
Other	21 (6.0)	21 (6.2)
Weight, mean (SD), kg	84.9 (21.2)	84.9 (21.2)
Range	47-156	47-156
Height, mean (SD), cm	165.0 (9.6)	165.0 (9.6)
Range	130-198	130-198
**RA clinical characteristics**		
Duration of RA, mean (SD), y	12.5 (9.7)	12.4 (9.6)
RA disease duration, n (%)		
< 3 y	42 (12.0)	42 (12.5)
3-4 y	39 (11.1)	38 (11.3)
5-10 y	93 (26.5)	87 (25.8)
> 10 y	177 (50.4)	170 (50.4)
**Prior rituximab treatment**		
No. of prior rituximab courses, n (%)		
0	306 (87.2)	293 (86.9)
1	24 (6.8)	24 (7.1)
2	21 (6.0)	20 (5.9)
Months since most recent rituximab course, n	45	44
Mean (SD)	6.8 (0.9)	6.7 (0.9)
Range	5-9	5-9
**Medication history**		
No. of prior anti-TNF agents, n (%)		
0	4 (1.1)	4 (1.2)
1	187 (53.3)	182 (54.0)
2	116 (33.0)	108 (32.0)
≥ 3	44 (12.5)	43 (12.8)
Prior non–anti-TNF biologic DMARDs		
Abatacept	17 (4.8)	16 (4.7)
Tocilizumab	12 (3.4)	12 (3.6)
MTX dose, mg/wk		
Mean (SD)	17.4 (4.7)	17.3 (4.7)
Median (range)	17.5 (8–25)	17.5 (8–25)
Oral steroid use, n (%)	150 (42.7)	143 (42.4)
Oral steroid dose, mg/d		
Mean (SD)	7.2 (3.4)	7.3 (3.4)
Median (range)	5 (2–25)	5 (2–25)

Anti-TNF, anti–tumor necrosis factor; DMARD, disease-modifying antirheumatic drug; IQR, interquartile range; MTX, methotrexate; RA, rheumatoid arthritis; SD, standard deviation. ^a^Of the 341 patients who received infusion 2, four were not included because medication volume was not recorded.

### Exposure to rituximab

Table 3 summarizes the total exposure to rituximab during the study and rituximab infusion duration by visit. Thirteen of the 351 patients who received Infusion 1 did not complete the 1000-mg infusion. The reasons included no medication volume recorded (n = 4), treatment modifications due to non-serious AEs (n = 7), medication error (n = 1), and an unknown reason (n = 1). The mean infusion time for Infusion 1 was 4.4 ± 0.3 hours, and approximately 12% (41/338) of patients receiving rituximab 1000 mg at Infusion 1 required more than 4.5 hours of infusion time.

**Table 3:** Exposure to rituximab during the study for all patients

	**Infusion 1**	**Infusion 2**	**Infusion 3**	**Infusion 4**
Received infusion	351^a^	341^a^	290^b^	278
Infusion at the faster rate	NA	337	289	278
Completed 1000 mg^c^	338	333	288	277
Total infusion hours				
n	338	333	288	277
Mean (SD)	4.4 (0.3)	2.0 (0.1)	2.1 (0.3)	2.0 (0.1)
Range	4.1-6.4	1.8-3.2	1.9-4.3	1.9-2.4
> 2.5 h	338 (100%)	5 (1.5%)	12 (4.2%)	0
> 4.5 h	41 (12.1%)	0	0	0

NA, not applicable; SD, standard deviation. ^a^Infusion rate and dose could not be determined for 4 patients because the site failed to record the infusion volume. Of these 4 patients, 3 at Day 168 and 2 at Day 182 received rituximab at the faster rate. ^b^Rituximab was administered at the standard infusion rate in 1 patient. ^c^Rituximab dose was derived from the sum of the volume received at each segment. To account for truncation errors, the derived dose of 970 mg or larger was considered as completed 1000 mg.

Four of the 337 patients who received Infusion 2 at the faster rate did not complete the full 1000-mg infusion due to non-serious AEs (n = 3) and an unknown reason (n = 1). One of the 289 patients who received Infusion 3 at the faster rate and 1 of the 278 patients who received Infusion 4 at the faster rate did not complete the full 1000-mg infusion due to a nonserious AE. The mean infusion times for Infusion 2, 3, and 4 were 2.0 ± 0.1, 2.1 ± 0.3, and 2.0 ± 0.1 hours, respectively. A small proportion of patients who received the full 1000-mg dose at Infusion 2 and 3 at the faster rate required more than 2.5 hours (1.5% [5/333] and 4.2% [12/288], respectively). No patients receiving Infusion 4 required more than 2.5 hours of infusion time.

### Safety findings

**Figure 3:**
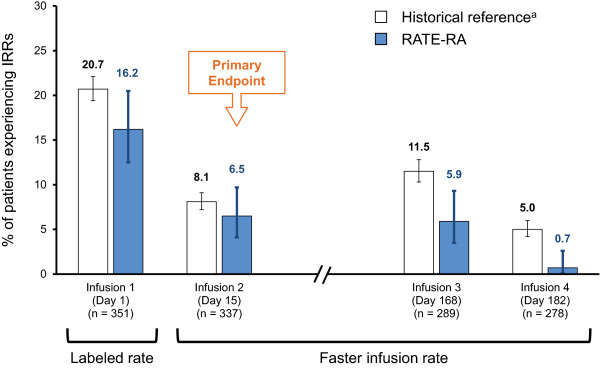
**IRRs during or within 24 hours of each rituximab infusion.** IRR, infusion-related reaction, RTX, rituximab. Error bars are 95% CI of the percentage. ^a^Weighted average of incidences reported in historical integrated data of phase 2 and phase 3 studies and open-label extension to date, adjusted for the proportion of patients with 0, 1, or 2 courses of prior rituximab.

The incidence of IRRs during or within 24 hours of each rituximab infusion is summarized in Figure 3. The proportion of patients who experienced IRRs decreased consistently with each infusion, and was consistent with or lower than historical clinical trial data. The incidence of IRRs during or within Infusion 1 was 16.2% (95% CI, 12.5% to 20.5%) and similar to the weighted historical incidence of 20.7% (95% CI, 19.4% to 22.1%). Of the 337 patients who received Infusion 2 at the faster rate, 22 patients (6.5% [95% CI, 4.1% to 9.7%]) experienced a total of 30 IRRs, with nausea (1.2%) and chills (0.9%) being the most common events. This incidence was similar to the weighted historical incidence of 8.1% (95% CI, 7.2% to 9.1%) at the standard infusion rate. The type and severity of events were also similar to the reference population. The incidence of IRRs for both Infusions 3 and 4 compared with the weighted historical incidences were as follows: 5.9% (95% CI, 3.5% to 9.3%) vs 11.5% (95% CI, 10.3% to 12.8%) for Infusion 3 and 0.7 (95% CI, 0.1% to 2.6%) vs 5.0% (95% CI, 4.2% to 6.0%) for Infusion 4. Ad hoc analyses showed no apparent trend between the maximum grade of IRRs at the first, second, or third infusion and the recurrence and grade of IRRs at subsequent infusions.

No serious IRRs or serious AEs (SAEs) were reported during or within 24 hours of any infusion (Table 4). All IRRs were CTC grade 1 or 2, except for 3 grade 3 IRRs associated with Infusion 1 (Day 1) in 2 patients (eye irritation and urticaria in 1 patient, throat irritation in the other patient) and 2 grade 3 IRRs associated with Infusion 2 (Day 15) (hypertension and headache in separate patients). Of the 2 patients who experienced grade 3 IRRs associated with Infusion 1, one withdrew prior to receiving Infusion 2; the other patient continued to receive the subsequent 3 infusions at the faster rate with no IRRs except for a grade 2 IRR at Infusion 3. Both patients with a grade 3 IRR at Infusion 2 received Infusions 3 and 4 at a faster rate with no IRRs. There were no grade 4 or fatal events. With a total of 904 rituximab infusions administered in 340 patients at the faster rate during Infusions 2, 3, and 4, the incidence of serious IRRs or SAEs associated with rituximab infused at the faster rate was < 0.9% (per-patient incidence) or < 0.33% (per-infusion incidence).

**Table 4:** Events during or within 24 hours of each rituximab infusion

**Events**	**Infusion 1 (N = 351) n (%)(95% CI)**^**a**^	**Infusion 2 (N = 337) n (%)(95% CI)**^**a**^	**Infusion 3 (N = 289) n (%)(95% CI)**^**a**^	**Infusion 4 (N = 278) n (%)(95% CI)**^**a**^
Serious IRRs	0 (0) (- to 0.8)	0 (0) (- to 0.9)	0 (0) (- to 1.0)	0 (0) (- to 1.1)
IRRs and other AEs	62 (17.7) (13.8 to 22.1)	24 (7.1) (4.6 to 10.4)	22 (7.6) (4.8 to 11.3)	5 (1.8) (0.6 to 4.1)
Serious AEs	0 (0) (- to 0.8)	0 (0) (- to 0.9)	0 (0) (- to 1.0)	0 (0) (- to 1.1)
CTC grade 3/4 AEs	2 (0.6) (0.1 to 2.0)	2 (0.6) (0.1 to 2.0)	0 (0) (0.1 to 2.1)	0 (0) (0.0 to 1.3)
Infusion stopped/slowed/interrupted^b^	43 (12.3) (9.0 to 16.1)	13 (3.9) (2.1 to 6.5)	19 (6.6) (4.0 to 10.1)	3 (1.1) (0.2 to 3.1)

AE, adverse event; CTC, Common Terminology Criteria; IRRs, infusion-related reactions. ^a^One-sided CI for serious events. ^b^Due to AE, medication error, or other reason such as bathroom break.

A total of 221 patients (63%) experienced at least one AE (inclusive of IRRs and other events) during the study. AEs reported in > 2% of patients included headache (5.7%), upper respiratory infection (5.4%), worsening RA (5.1%), pruritus (4.3%), sinusitis (4.0%), urinary tract infection (3.7%), throat irritation (3.4%), arthralgia (3.1%), nausea (3.1%), flushing (2.6%), cough (2.3%), dizziness (2.3%), ear pruritus (2.3%), and rash (2.3%). A total of 33 SAEs were reported in 30 patients (8.5%) during the study, with a per-100 patient-year rate of 16.0 (95% CI, 11.3 to 22.4). Of the 33 SAEs, 3 (pneumonia, sepsis, and septic shock) were considered by investigators to be related to rituximab treatment. Ten patients (2.8%) had a total of 12 serious infectious events (defined as serious infections or events treated with IV antibiotics) reported during the study, with a per-100 patient-year rate of 5.8 (95% CI, 3.3 to 10.2). These rates are consistent with what has been previously reported for rituximab in RA [1,3]. There were no deaths in this study.

### Subgroup analysis by prior rituximab experience

As expected, rituximab-naive patients had a higher incidence of IRRs compared with rituximab-experienced patients at Infusion 1 (17.3% [95% CI, 13.3% to 22.0%] vs 8.9% [95% CI, 2.5% to 21.2%], respectively). The incidence of IRRs was similar between rituximab-naive and -experienced patients during or within 24 hours of Infusion 2 (6.5% [95% CI, 3.9% to 9.9%] vs 6.8% [95% CI, 1.4% to 18.7%]), Infusion 3 (6.4% [95% CI, 3.7% to 10.1%] vs 2.6% [95% CI, 0.1% to 13.8%]), and Infusion 4 (0.4% [95% CI, 0.0% to 2.3] vs 2.9% [95% CI, 0.1% to 14.9%]).

## Discussion

In this prospective, open-label study, the reported incidence of IRRs for the first rituximab infusion of course 1 was 16.2%. When rituximab was infused at a faster rate over a 2-hour period during the second infusion of Course 1 and the first and second infusions of Course 2, the incidence of IRRs was 6.5% (primary endpoint), 5.9%, and 0.7%, respectively. These observed incidences were no greater than the weighted historical incidences of IRRs when rituximab was infused at the standard rate, demonstrating that rituximab can be administered at the faster rate at the second and subsequent infusions without increasing the risk of IRRs in patients with RA. In addition, the type and severity of IRRs were similar to the reference population. There were no serious IRRs or SAEs during or within 24 hours of any infusion during the 2 courses of treatment. Subgroup analysis indicated that rates of IRR were similar regardless of prior rituximab-exposure. Overall, the safety results such as SAEs and serious infections were consistent with those reported in 2 rituximab pivotal trials in patients who were anti-TNF inadequate responders [1,3].

The safety and feasibility of shorter rituximab infusion times have been evaluated in several published studies [9-17]. In general, the safety of the faster infusion has been reported in previously published studies to be comparable to that of the standard 4.25 hour infusion, and the results of the present study confirm these findings. Of note, in the oncology setting, a study called RATE utilized a similar study design as the present RATE-RA study [18]. These results supported a label change that provides for an option for a faster infusion schedule of rituximab from cycle 2 onward for patients with NHL who tolerated their first rituximab infusion administered at the standard rate. Given that patients with RA have a lower baseline B cell count compared with patients with lymphoma and generally tolerate the infusion better, patients in RATE-RA were expected to and did tolerate the faster infusion rate, consistent with the results from the oncology setting.

This was the first, large, prospective study to evaluate the safety of a faster rate of rituximab in patients with RA. External control incidences were calculated based on integrated data from the rituximab RA clinical development program that used pooled data of all patients exposed to rituximab (all-exposure population). The expected control incidence was calculated based on historical incidences weighted to account for patients with previous rituximab treatment. These reference rates provide a more robust comparison than IRR incidences found in the available literature, which are limited by small or dissimilar patient populations. Because this study assessed the safety of rapidly infused rituximab against the standard rituximab infusion rate, the lack of active comparators might be considered a limitation.

## Conclusions

In conclusion, this study demonstrated that rituximab can be administered at the faster infusion rate at the second and subsequent infusions without increasing the risk or severity of IRRs in patients with RA. As shorter infusion times can improve not only patient satisfaction but also infusion center efficiency, the results of this study provide clinically relevant information for both health care providers and patients.

## Competing interests

CP has received speaker fees from Genentech, Abbvie, Pfizer, BMS; MG has received research grants from Genentech/Roche; JK has received research grants from Genentech, Pfizer, UCB and Novartis and has received consulting fees from BMS, Genentech, Pfizer, Lilly; NG has received research grants and consulting fees from Genentech/Roche; WRigby has received consulting fees from Roche Pharmaceuticals; SZ, CC and WReiss are employees of Genentech Inc; BJ is an employee of Hoffmann-La Roche Ltd.

## Authors’ contributions

CP, SZ, BJ and WReiss conceived and designed the research. All authors made substantial contributions to the analysis and/or interpretation of the data and to drafting and revising the manuscript. CC performed the statistical analysis. All authors read and approved the final manuscript.

## Pre-publication history

The pre-publication history for this paper can be accessed here:

http://www.biomedcentral.com/1471-2474/15/177/prepub

## References

[B1] Cohen SB Emery P Greenwald MW Dougados M Furie RA Genovese MC Keystone EC Loveless JE Burmester GR Cravets MW Hessey EW Shaw T Totoritis MC REFLEX Trial Group Rituximab for rheumatoid arthritis refractory to anti-tumor necrosis factor therapy: results of a multicenter, randomized, double-blind, placebo-controlled, phase III trial evaluating primary efficacy and safety at twenty-four weeks Arthritis Rheum 2006 54 9 2793 2806 10.1002/art.22025 16947627

[B2] Keystone E Emery P Peterfy CG Tak PP Cohen S Genovese MC Dougados M Burmester GR Greenwald M Kvien TK Williams S Hagerty D Cravets MW Shaw T Rituximab inhibits structural joint damage in patients with rheumatoid arthritis with an inadequate response to tumour necrosis factor inhibitor therapies Ann Rheum Dis 2009 68 2 216 221 10.1136/ard.2007.085787 18388156

[B3] Mease PJ Cohen S Gaylis NB Chubick A Kaell AT Greenwald M Agarwal S Yin M Kelman A Efficacy and safety of retreatment in patients with rheumatoid arthritis with previous inadequate response to tumor necrosis factor inhibitors: results from the SUNRISE trial J Rheumatol 2010 37 5 917 927 10.3899/jrheum.090442 20194448

[B4] Emery P Deodhar A Rigby WF Isaacs JD Combe B Racewicz AJ Latinis K Abud-Mendoza C Szczepanski LJ Roschmann RA Chen A Armstrong GK Douglass W Tyrrell H Efficacy and safety of different doses and retreatment of rituximab: a randomised, placebo-controlled trial in patients who are biological naive with active rheumatoid arthritis and an inadequate response to methotrexate (Study Evaluating Rituximab’s Efficacy in MTX iNadequate rEsponders (SERENE)) Ann Rheum Dis 2010 69 9 1629 1635 10.1136/ard.2009.119933 20488885 PMC2938895

[B5] Rituxan(r) [package insert] 2013 South San Francisco, CA: Genentech, Inc

[B6] Chung CH Managing premedications and the risk for reactions to infusional monoclonal antibody therapy Oncologist 2008 13 6 725 732 10.1634/theoncologist.2008-0012 18586928

[B7] Atmar J Review of the safety and feasibility of rapid infusion of rituximab J Oncol Pract 2010 6 2 91 93 10.1200/JOP.200001 20592783 PMC2835489

[B8] van Vollenhoven RF Emery P Bingham CO Keystone EC 3rd Fleischmann RM Furst DE Tyson N Collinson N Lehane PB Long-term safety of rituximab in rheumatoid arthritis: 9.5-year follow-up of the global clinical trial programme with a focus on adverse events of interest in RA patients Ann Rheum Dis 2013 72 9 1496 1502 10.1136/annrheumdis-2012-201956 23136242 PMC3756452

[B9] Sehn LH Donaldson J Filewich A Fitzgerald C Gill KK Runzer N Searle B Souliere S Spinelli JJ Sutherland J Connors JM Rapid infusion rituximab in combination with corticosteroid-containing chemotherapy or as maintenance therapy is well tolerated and can safely be delivered in the community setting Blood 2007 109 10 4171 4173 10.1182/blood-2006-11-059469 17244675

[B10] Tuthill M Crook T Corbet T King J Webb A Rapid infusion of rituximab over 60 min Eur J Haematol 2009 82 4 322 325 10.1111/j.1600-0609.2009.01215.x 19220420

[B11] Can M Alibaz-Oner F Yilmaz-Oner S Atagunduz P Inanc N Direskeneli H Accelerated infusion rates of rituximab are well tolerated and safe in rheumatology practice: a single-centre experience Clin Rheumatol 2013 32 1 87 90 10.1007/s10067-012-2094-1 23053686

[B12] Faraawi R Roth K Experience with accelerated rituximab infusion for rheumatoid arthritis in a single community practice Ann Rheum Dis 2010 69 Suppl 3 383

[B13] Bukh G Larsen S Rasmussen SS Molgard MK Hansen M Accelerated infusion rate of rituximab for rheumatoid arthritis is well tolerated and safe Arthritis Rheum 2008 58 9, Suppl. S857 S858

[B14] Bukh G Larsen SS Rasmussen MS Very fast infusion-rate of rituximab for rheumatoid arthritis is well tolerated and safe Ann Rheum Dis 2011 70 Suppl III 754

[B15] Larsen JL Jacobsen S Rapid infusion with rituximab: short term safety in systemic autoimmune diseases Rheumatol Int 2013 33 2 529 533 10.1007/s00296-011-2208-0 22068354

[B16] Schoeffel DA Henn SM Goedde A Simplified treatment protocol of rituximab in rheumatoid arthritis Ann Rheum Dis 2008 67 Suppl II 337

[B17] Larsen JL Jacobsen S Rapid infusion with rituximab: short term safety in systemic autoimmune diseases Ann Rheum Dis 2010 69 Suppl III 731 10.1007/s00296-011-2208-0 22068354

[B18] Dakhil S Hermann R Chai A Hurst D Fine G Richards P Final results of a single arm phase Ill multicenter, open-label study of rituximab administered by faster infusion in patients with previously untreated diffuse large B-cell (DLBCL) or follicular non-Hodgkin’s lymphoma (FL) [abstract] Presented at: 53rd American Society of Hematology (ASH) Annual Meeting and Exposition; December 10–13, 2011; San Diego, CA Abstract #2703

